# Dual-task standing in older adults with mild cognitive impairment: postural control complexity, prefrontal activation, and cognitive task performance

**DOI:** 10.3389/fnins.2026.1885763

**Published:** 2026-09-17

**Authors:** Xinru Li, Mian Zhou, Yvxi Zhang, Guocai Xu, Min Mao, Dewei Mao, Jiangna Wang, Wei Sun

**Affiliations:** 1 Graduate School of Education, Shandong Sport University, Jinan, Shandong, China; 2 Rehabilitation Medicine Department, Weishan People’s Hospital, Jining, Shandong, China; 3 Shenyang Sport University, Shenyang, Liaoning, China; 4 Department of Sport and Recreation, Technological and Higher Education Institute of Hong Kong, Hong Kong, China; 5 Division of Physical Education, The Chinese University of Hong Kong, Shenzhen, China; 6 College of Sports and Health, Shandong Sport University, Jinan, Shandong, China

**Keywords:** fNIRS, mild cognitive impairment, postural control, sample entropy, standing balance

## Abstract

**Background:**

This study aimed to quantify prefrontal cortex (PFC) activation and postural control complexity in older adults with mild cognitive impairment (MCI) during single-task standing and dual-task standing balance conditions and to investigate the associations between these measures.

**Methods:**

This secondary analysis included 21 older adults with MCI (71.19 ± 3.36 years) and 19 cognitively intact controls (70.16 ± 4.54 years) from a previously reported cohort. Outcomes included cognitive performance, sample entropy of COP displacement (SampEn-COP), PFC activation across four bilateral functional regions, and exploratory correlations between PFC activation and SampEn-COP.

**Results:**

A significant group effect was observed for SampEn-COPml, with lower values in the MCI group than in the control group (*p* = 0.002, η^2^_p_ = 0.217, q = 0.007). SampEn-COPap was significantly higher under the dual-task condition than under the single-task condition (*p* = 0.005, η^2^_p_ = 0.187, q = 0.016). Group × Task interactions were observed at the uncorrected level in the dorsolateral prefrontal cortex (DLPFC; *p* = 0.041, η^2^_p_ = 0.106, q = 0.073) and orbitofrontal cortex (OFC; *p* = 0.039, η^2^_p_ = 0.107, q = 0.073). The MCI group also showed lower cognitive accuracy and response rates than the control group across task conditions (both q < 0.01). An exploratory negative correlation between OFC activation and SampEn-COPap was observed in the MCI group under the single-task condition (*r* = −0.469, *p* = 0.032, q = 0.256).

**Conclusion:**

Older adults with MCI showed a less complex medial–lateral postural control pattern than cognitively intact older adults. During dual-task standing, changes in postural complexity were accompanied by preliminary evidence of task-related increases in DLPFC and OFC activation in the MCI group, suggesting greater prefrontal involvement in managing concurrent cognitive and postural demands.

## 1 Introduction

Mild cognitive impairment (MCI) represents a transitional stage between normal aging and dementia, characterized by objective cognitive decline that does not yet impair daily functioning (Liu et al., 2020; Petersen et al., 2018). The prevalence of older adults with MCI ranges from 6.7 to 25.2% (Jongsiriyanyong and Limpawattana, 2018). The risk of falls in older adults with MCI is about twice that in cognitively intact older adults (Lach et al., 2017).

MCI constitutes a clinically significant risk factor for postural instability and falls in older adults. Postural control is a complex process requiring engagement of cognitive resources, and declines in cognitive function may impair balance control, thereby increasing the risk of falls (Lacour et al., 2008). During dual-task performance, competition for limited attentional resources may affect both cognitive performance and postural control (Lacour et al., 2008; Liu et al., 2020). Conventional center of pressure (COP) measures, such as COP displacement, quantify the magnitude of postural sway, with larger values generally reflecting greater sway and possibly indicating lower postural stability (Prieto et al., 1996). However, these conventional measures primarily characterize the magnitude of postural sway and provide limited information about the temporal organization of postural control.

Beyond these conventional measures of postural sway, sample entropy is a nonlinear measure of time-series regularity that has been widely used to characterize the complexity of COP dynamics. Sample entropy of COP (SampEn-COP) is considered more sensitive to postural control strategies than traditional linear COP measures (Kędziorek and Błażkiewicz, 2020; Cofré Lizama et al., 2024). Lower SampEn-COP has been reported in older adults with a history of lateral ankle sprain and was interpreted as reflecting a more rigid and less adaptable pattern of postural control (Terada et al., 2018). Older fallers have also been reported to show generally higher anterior–posterior but lower medial–lateral SampEn-COP values than non-fallers (Montesinos et al., 2018). Greater SampEn-COP values indicate less regular and more complex COP fluctuations, while lower values reflect more regular and less complex fluctuations (Cofré Lizama et al., 2024; Kędziorek and Błażkiewicz, 2020). Lower postural control complexity has previously been reported in older adults with MCI during standing. Reduced anterior–posterior and medial–lateral complexity was observed across single-task standing and dual-task standing involving visual search, as quantified by multiscale entropy (Zhou et al., 2023), while lower medial–lateral SampEn was found during standing on foam with the eyes closed (Thammachat et al., 2026). However, neither study examined PFC activation alongside postural control complexity.

Dual-task interference may further challenge postural control in older adults with MCI. Dual-task paradigms are commonly used to examine how concurrent cognitive demands influence postural performance (Boisgontier et al., 2013). Serial subtraction by threes has been used during dual-task standing to impose a sustained cognitive load while allowing concurrent assessment of cognitive performance (Kahya et al., 2022). Task-related changes in prefrontal cortex (PFC) activation have been reported during dual-task standing and walking in older adults, consistent with a role for the PFC in meeting concurrent cognitive and postural demands (St George et al., 2021; Baek et al., 2023). Functional near-infrared spectroscopy (fNIRS) has been widely used to assess PFC activity, with changes in oxyhemoglobin concentration (ΔHbO_2_) during task performance commonly used as an index of cortical activation during both cognitive and balance tasks in dual-task paradigms (Herold et al., 2017; St George et al., 2021). The relationship between PFC activation and postural control complexity under dual-task conditions in older adults with MCI remains unclear.

Building on the conventional COP and PFC activation analyses reported by Xu et al. (2024), the present secondary analysis aimed to examine postural control complexity, task-related PFC activation, and cognitive task performance during the single-task and dual-task conditions. This study may help to characterize the neural and behavioral features of postural control in older adults with MCI and inform future research on targeted rehabilitation strategies. We hypothesized that older adults with MCI would exhibit lower postural control complexity, lower PFC activation and poorer cognitive task performance than cognitively intact older adults, particularly under the dual-task condition. Associations between postural control complexity and PFC activation were examined as exploratory analyses based on the primary findings.

## 2 Materials and methods

### 2.1 Participants

This study was a secondary analysis of data obtained from the same participant cohort and experimental recordings as those reported by Xu et al. (2024).

Sample size for the original study was determined *a priori* using G*Power 3.1. Based on a previously reported condition effect on HbO_2_ (η^2^_p_ = 0.289; Marusic et al., 2019), with α = 0.05 and power = 0.80, the minimum required total sample size was 22 participants. A total of 40 participants were recruited. The MCI group included 21 older adults who met the study criteria for MCI (age 65–76 years, mean age 71.19 ± 3.36 years), and the control group included 19 cognitively intact older adults (age 65–76 years, mean age 70.16 ± 4.54 years).

Participants were classified into the MCI group according to the criteria used for the original cohort, which included subjective memory complaints, cognitive impairment assessed using the MMSE and MoCA, preserved independence in daily functioning, absence of clinical dementia, and a Clinical Dementia Rating score of 0.5 (Petersen et al., 2018; Xu et al., 2024). The MoCA was used as a standardized cognitive screening tool, and a score < 26 was required for inclusion in the MCI group. Inclusion criteria were defined as follows (Montero-Odasso et al., 2012; Xu et al., 2024): MCI group: (a) community-dwelling adults aged ≥ 65 years; (b) meeting the MCI criteria described above; (c) MoCA score < 26; and (d) independent ambulation without assistive devices. Control group: (a) community-dwelling adults aged ≥ 65 years; (b) MoCA score ≥ 26; (c) no documented cognitive impairment or functional impairment; and (d) independent ambulation without assistive devices. Exclusion criteria included: (a) diagnosed neurological disorders associated with motor deficits; (b) musculoskeletal abnormalities or other conditions affecting gait mechanics; (c) severe uncorrected visual or auditory deficits; and (d) use of psychotropic medications that could affect motor performance or the presence of active major depression.

The MCI and control groups demonstrated comparable baseline characteristics with no significant differences in age, height, body weight, or educational attainment (Table 1).

**Table 1:** Participant characteristics (mean ± SD).

Characteristic	Control group (*n* = 19)	MCI group (*n* = 21)	*p* value
Age (years)	70.16 ± 4.54	71.19 ± 3.36	0.415
Sex (male/female)	10/9	9/12	0.536
Height (cm)	163.53 ± 6.63	159.71 ± 7.29	0.055
Weight (kg)	64.46 ± 8.07	63.61 ± 10.04	0.771
Years of education (years)	6.74 ± 3.40	6.05 ± 3.31	0.520
MoCA	26.47 ± 0.90	18.33 ± 2.97	< 0.001

### 2.2 Experimental design

A cross-sectional mixed-design study was used. Participants were classified into two groups (MCI group and control group) and tested under two randomly ordered standing conditions (single-task and dual-task). Each participant attended one laboratory session lasting approximately 90 min. Force measuring platform and fNIRS data were recorded simultaneously during 60-s standing trials, with three trials completed under each condition. The outcomes analyzed in the present study included SampEn-COP, concentration changes in oxyhemoglobin (ΔHbO_2_, μmol/L) in PFC, and cognitive task performance. The task order was randomized using a computer-based random number generator to ensure unpredictability in the sequence. All data preprocessing and outcome extraction were performed by assessors blinded to participant group, and group labels were linked only after these procedures were completed for statistical analysis.

The present analysis examined the temporal complexity of COP dynamics, PFC activation, and cognitive task performance. The original study protocol was approved by the Ethics Committee of Shandong Sport University (Approval No. 2021006) and was conducted in accordance with the Declaration of Helsinki. Written informed consent was obtained from all participants prior to study enrollment. The original study was prospectively registered with the Chinese Clinical Trial Registry (ChiCTR2100044221).

### 2.3 Measures and procedures

#### 2.3.1 Balance tests

The standing balance control task was performed in the Sports Biomechanics Laboratory. Participants were required to perform the following two conditions: (1) Single-task standing: Participants stood barefoot at the center of the force measuring platform, hands on hips, feet parallel and 20 cm apart (Shin et al., 2011). Participants were instructed to stand upright with the upper body kept straight and their gaze fixed forward. Each trial lasted 60 s and ended when participants heard the “stop” command. (2) Dual-task standing: This task required participants to perform a cognitive task while maintaining standing balance. The cognitive task involved continuously subtracting 3 from a random number between 400 and 500 that was verbally provided by the examiner at the beginning of each trial (Marusic et al., 2019; Bishnoi and Hernandez, 2021). Serial subtraction by threes requires sustained attention and working memory processing and is commonly used as a concurrent cognitive task in dual-task studies (Fallahtafti et al., 2021). Participants were instructed to perform the calculations aloud as accurately as possible throughout the entire 60-s dual-task standing period. For single-task cognitive performance, participants completed the same serial subtraction task while seated for three 60-s trials. The order of the single-task cognitive condition and the two standing conditions was randomized, and three trials were completed for each standing condition.

The fNIRS probes were positioned over the prefrontal scalp according to the international 10–20 system. Before each standing task, participants completed a 60-s seated rest period with their eyes closed. Participants then transitioned from sitting to standing; this postural transition phase was not included in the analysis. To minimize the influence of transient systemic and hemodynamic fluctuations associated with postural change, participants were required to stand quietly for a short stabilization period (~15 s) before the onset of the standing task, allowing cardiovascular and cerebral hemodynamics to stabilize. At task onset, data acquisition from the force measuring platform was initiated, and a marker was simultaneously triggered in the fNIRS system, ensuring precise temporal alignment between force and fNIRS data. The 60-s period beginning at the task onset marker was defined as the standing task period, and the 5 s immediately preceding task onset served as the quiet-standing baseline (Figure 1).

**Figure 1:**
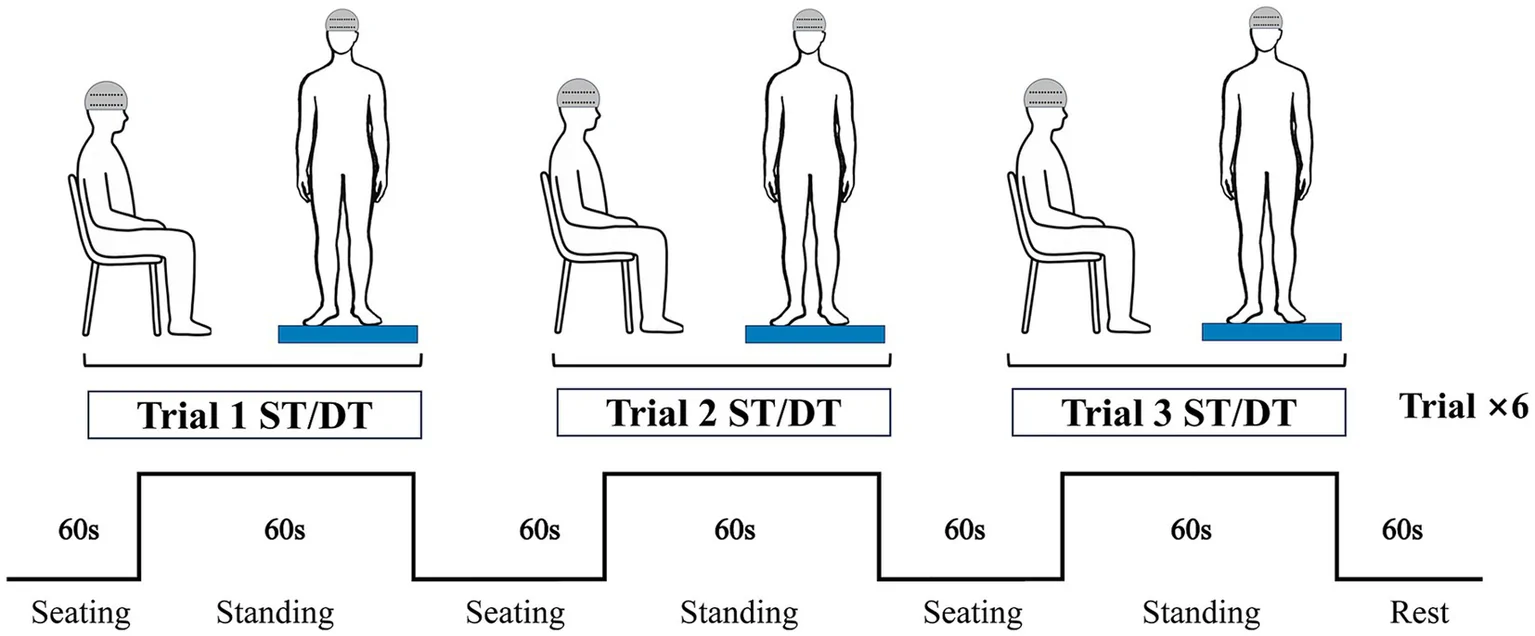
Testing procedure and device.

COP displacement data were collected during the balance task using a force measuring platform (Kistler 9281CA, 60 cm × 90 cm × 10 cm) at a sampling frequency of 1,000 Hz for 60 s.

#### 2.3.2 Functional near-infrared spectroscopy test

A portable near-infrared imaging system (LIGHTNIRS, Shimadzu Corp., Kyoto, Japan) was used to measure the PFC hemodynamic responses during upright posture maintenance, with data acquired at a frequency of 13.3 Hz. The device consists of 16 optodes, including 8 light emitters (LEDs; wavelengths: 780/805/830 nm) and 8 light detectors, with a 30 mm spacing between the emitters and the detectors, and a total of 22 measurement channels. According to Brodmann areas (BAs), the 22 PFC channels were organized into 4 regions of interest (ROIs). Probe coordinate information was collected using a 3D digitizer (FASTRAK, Polhemus, Vermont, USA) to determine the Montreal Neurological Institute (MNI) coordinates of the prefrontal channels.

Brain activation analyses were conducted in four predefined prefrontal regions: the frontopolar cortex (FPC) included Channels 2, 3, 4, 5, 6, 11, 12, 13, 19 and 21. The dorsolateral prefrontal cortex (DLPFC) included Channels 9, 14, 16 and 22. The ventrolateral prefrontal cortex (VLPFC) included Channels 1, 7, 8 and 15. The orbitofrontal cortex (OFC) included Channels 10, 17, 18 and 20 (Figure 2).

**Figure 2:**
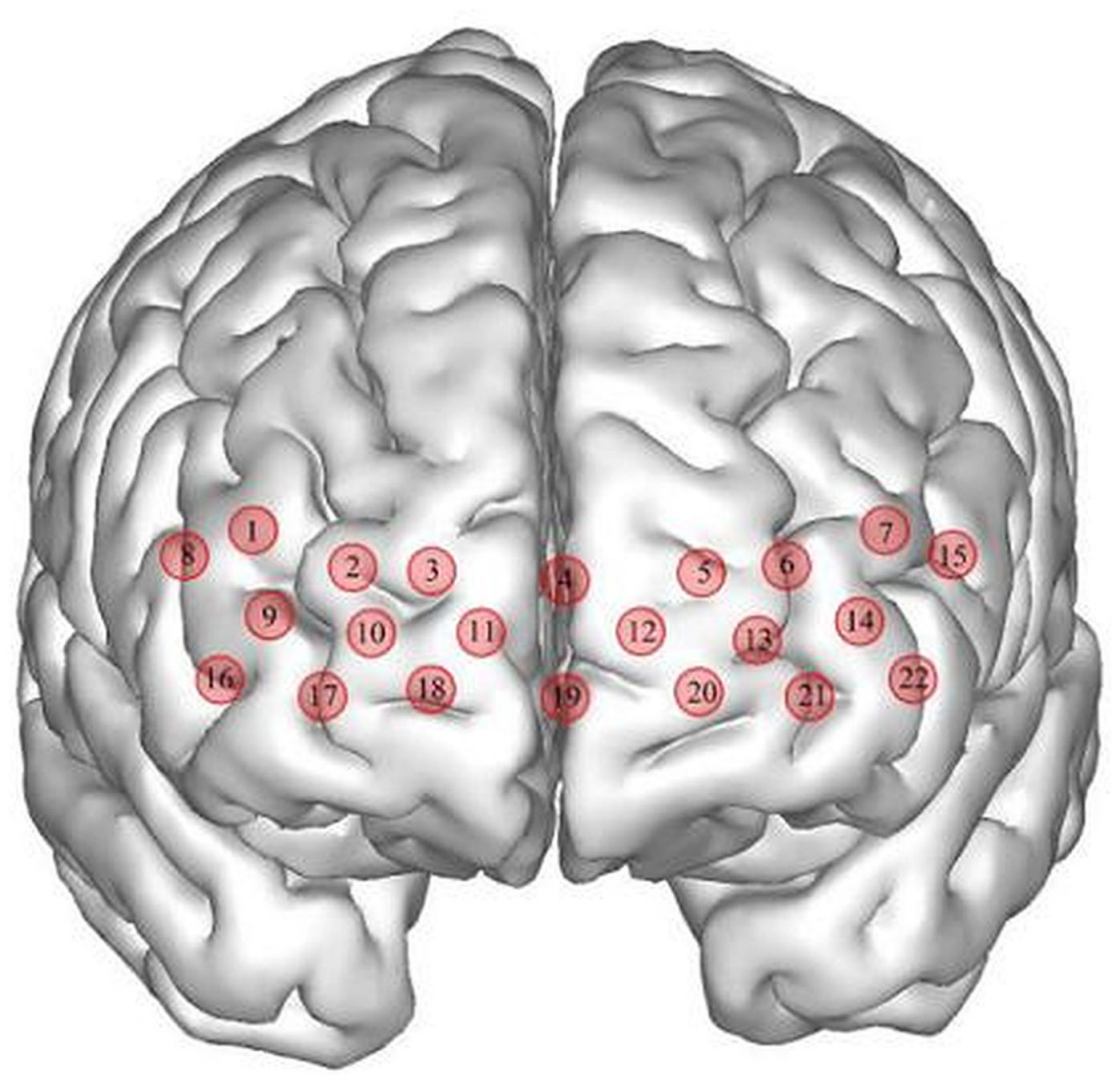
Distribution map of 22 channels.

### 2.4 Data processing

#### 2.4.1 Balance data processing

Prior to entropy analysis, COP signals were exported from BioWare after low-pass filtering at 10 Hz (Fischer et al., 2023). The COP signals were downsampled to 100 Hz using an anti-aliasing filter. The complete 60-s segments were used for SampEn analysis without interpolation. The anterior–posterior (AP) and medial–lateral (ML) COP displacement time series were centered by subtracting their respective trial means. Resultant COP displacement, hereafter referred to as COPtotal, was then calculated as the Euclidean distance from the trial-specific mean COP position (Prieto et al., 1996; Isableu et al., 2017):

$$\mathrm{CO}P_{\text{resultant}\;(t)}=\sqrt{{\left[\mathrm{CO}P_{\left(\mathrm{AP})(t\right)}-\bar{\left(\mathrm{CO}P_{\mathrm{AP}}\right)}\right]}^{2}+{\left[\mathrm{CO}P_{\left(\mathrm{ML})(t\right)}-\bar{\left(\mathrm{CO}P_{\mathrm{ML}}\right)}\right]}^{2}}$$

SampEn (Richman and Moorman, 2000; Ruiz-Gómez et al., 2018) was calculated for the three COP displacement directions time series using a custom Python script with the EntropyHub package. SampEn was computed with an embedding dimension of m = 2, a time delay of τ = 1, and a tolerance of *r* = 0.2 × the standard deviation of each time series.

Three metrics were selected: the sample entropy of COP displacement in the anterior–posterior direction (SampEn-COPap), the sample entropy of COP displacement in the medial-lateral direction (SampEn-COPml), and the sample entropy of resultant COP displacement (SampEn-COPtotal). These metrics were used to evaluate the time series complexity of the anterior–posterior, medial–lateral, and resultant COP displacement time series, with higher values indicating greater irregularity and lower temporal predictability.

#### 2.4.2 fNIRS data processing

LIGHTNIRS data were imported and processed using the Homer2 toolbox in MATLAB (R2024a, MathWorks Inc., Natick, MA, USA). The fNIRS signals were processed in optical density space. Motion artifacts were identified separately for each channel using hmrMotionArtifactByChannel, with a motion detection window of 0.5 s, a temporal mask of 1.0 s, a standard deviation threshold of 20, and an amplitude threshold of 0.10. Motion artifact segments were then corrected using spline interpolation (hmrMotionCorrectSpline; spline parameter = 0.99). The corrected signals were then band-pass filtered (0.01–0.10 Hz) to attenuate low-frequency signal drift and physiological noise (Zu et al., 2023). The filtered optical density signals were converted to hemoglobin concentration changes via the modified Beer–Lambert law, yielding time series for oxyhemoglobin (HbO_2_) and deoxyhemoglobin (HbR). HbO_2_ signals were selected for analysis because they generally exhibit a larger task-related amplitude and a higher signal-to-noise ratio than HbR.

For each channel, ΔHbO_2_ was calculated as the difference between the mean HbO_2_ concentrations during the 60-s task and the 5-s quiet-standing baseline immediately preceding task onset. Channel-level ΔHbO_2_ values were averaged within each ROI and across valid trials for each participant and task condition.

#### 2.4.3 Cognitive task performance processing

The serial subtraction task was performed both alone and during the dual-task condition. Cognitive task performance was assessed using accuracy and response rate. Accuracy was calculated as:

$$\text{Accurac}y_{i,c}(\%)=\frac{\sum_{j=1}^{N}\text{Correc}t_{i,c,j}}{\sum_{j=1}^{N}(\text{Correc}t_{i,c,j}+\text{Incorrec}t_{i,c,j})}\times 100\%$$

where total responses were defined as the sum of correct and incorrect responses and N denotes the number of valid trials. Response rate was calculated as:

$$\text{Response}\;\mathrm{rat}e_{i,c}=\frac{\sum_{j=1}^{N}\text{Tota}l_{i,c,j}}{\sum_{j=1}^{N}\text{Duratio}n_{i,c,j}}\times 60$$

and was expressed as responses per minute. Cognitive dual-task cost (DTC) was calculated for both accuracy and response rate using the following formulas:

$$\text{Accuracy}\;\mathrm{DTC}(\%)=\frac{\text{Accurac}y_{ST_{\mathrm{cog}}}-\text{Accurac}y_{DT_{\mathrm{cog}}}}{\text{Accurac}y_{ST_{\mathrm{cog}}}}\times 100\%$$

$$\text{Response rate}\;\mathrm{DTC}(\%)=\frac{\text{Response}\;\mathrm{rat}e_{ST_{\mathrm{cog}}}-\text{Response}\;\mathrm{rat}e_{DT_{\mathrm{cog}}}}{\text{Response}\;\mathrm{rat}e_{ST_{\mathrm{cog}}}}\times 100\%$$

where $ST_{\mathrm{cog}}$ represents the single-task cognitive condition and $DT_{\mathrm{cog}}$ represents the dual-task condition. Positive DTC values indicated lower accuracy or response rate under the dual-task condition than under the single-task cognitive condition, whereas negative values indicated higher accuracy or response rate under the dual-task condition.

### 2.5 Statistical analyses

Statistical analyses were performed using SPSS 26.0 (IBM SPSS, Armonk, NY, USA). For each participant, the average of three repeated trials under each condition was used for analysis. For each variable, values exceeding ± 3 standard deviations from the mean were considered outliers and excluded from the corresponding analyses. All variables were presented as mean ± standard deviation. The Shapiro–Wilk test was conducted to assess normality of the data. The two-way mixed-design analysis of variance (ANOVA) was conducted, with group (MCI vs. control) as the between-subject factor and task condition (single-task vs. dual-task) as the within-subject factor, to examine the main effects of group, task, and their interaction. If significant interaction effects were present, Bonferroni *post-hoc* analyses were performed for multiple comparisons. Exploratory Pearson correlation analyses were conducted to explore the relationships between ΔHbO_2_ in the DLPFC or OFC and SampEn-COPap. Accuracy DTC and response rate DTC were compared between groups using independent-samples t tests. The Benjamini–Hochberg FDR procedure was applied within each set of related outcomes to correct for multiple comparisons. Statistical significance was set at *p* < 0.05 and q < 0.05 indicated significance after FDR correction.

Partial eta squared (η^2^_p_) was used to represent the effect size of main effects and interactions in ANOVA. Cohen’s d (d) was used to represent the effect size of *post-hoc* analysis and correlation coefficient (r) was reported for correlation analyses.

## 3 Results

### 3.1 Cognitive task performance

Accuracy showed no significant Group × Task interaction (*p* = 0.472, η^2^_p_ = 0.014). The group main effect indicated that participants with MCI had lower accuracy than controls (*p* < 0.001, η^2^_p_ = 0.401, q < 0.001), whereas the task main effect indicated that accuracy was lower under the dual-task condition than under the single-task condition (*p* = 0.007, η^2^_p_ = 0.177, q = 0.014).

Response rate showed a significant Group × Task interaction (*p* = 0.018, η^2^_p_ = 0.139, q = 0.036). A significant group main effect was also observed, with lower response rates in the MCI group than in the control group (*p* = 0.007, η^2^_p_ = 0.175, q = 0.007). Bonferroni-adjusted *post hoc* comparisons indicated that response rate was significantly higher under the dual-task condition than under the single-task condition in the control group (95% CI: 0.59 to 2.09, *p* = 0.001, d = 0.86), whereas no significant difference was observed between task conditions in the MCI group.

Accuracy DTC did not differ significantly between groups (95% CI for the between-group difference: −8.03 to 3.92, *p* = 0.490, d = −0.22). Response rate DTC also did not differ significantly between groups (95% CI for the between-group difference: −26.71 to 6.11, *p* = 0.208, d = −0.39). Complete cognitive task performance results and BH-FDR q values are provided in Supplementary Table S1.

### 3.2 SampEn-COP measures

Two-way mixed-design ANOVA revealed a Group × Task interaction that was significant at the uncorrected level for SampEn-COPap (*p* = 0.047, η^2^_p_ = 0.100, q = 0.140), whereas no significant interaction effects were found for SampEn-COPml (*p* = 0.638, η^2^_p_ = 0.006) or SampEn-COPtotal (*p* = 0.124, η^2^_p_ = 0.061). Bonferroni-adjusted *post hoc* analyses showed that, in the control group, SampEn-COPap was significantly higher under the dual-task condition than under the single-task condition (95% CI: 0.0075 to 0.0288, *p* = 0.001, d = 0.69), whereas no significant task difference was observed in the MCI group (*p* = 0.518).

For the main effects, significant task effects were observed for SampEn-COPap (*p* = 0.005, η^2^_p_ = 0.187, q = 0.016), with higher values under the dual-task condition than under the single-task condition. No significant task effects were found for SampEn-COPml (*p* = 0.510, η^2^_p_ = 0.012) or SampEn-COPtotal (*p* = 0.156, η^2^_p_ = 0.052). A significant group effect was observed for SampEn-COPml (*p* = 0.002, η^2^_p_ = 0.217, q = 0.007), whereas the group effect for SampEn-COPtotal was observed only at the uncorrected level (*p* = 0.046, η^2^_p_ = 0.101, q = 0.069), with lower values in the MCI group than in the control group. Complete ANOVA results and BH-FDR q values are provided in Supplementary Table S2 (Figure 3).

**Figure 3:**
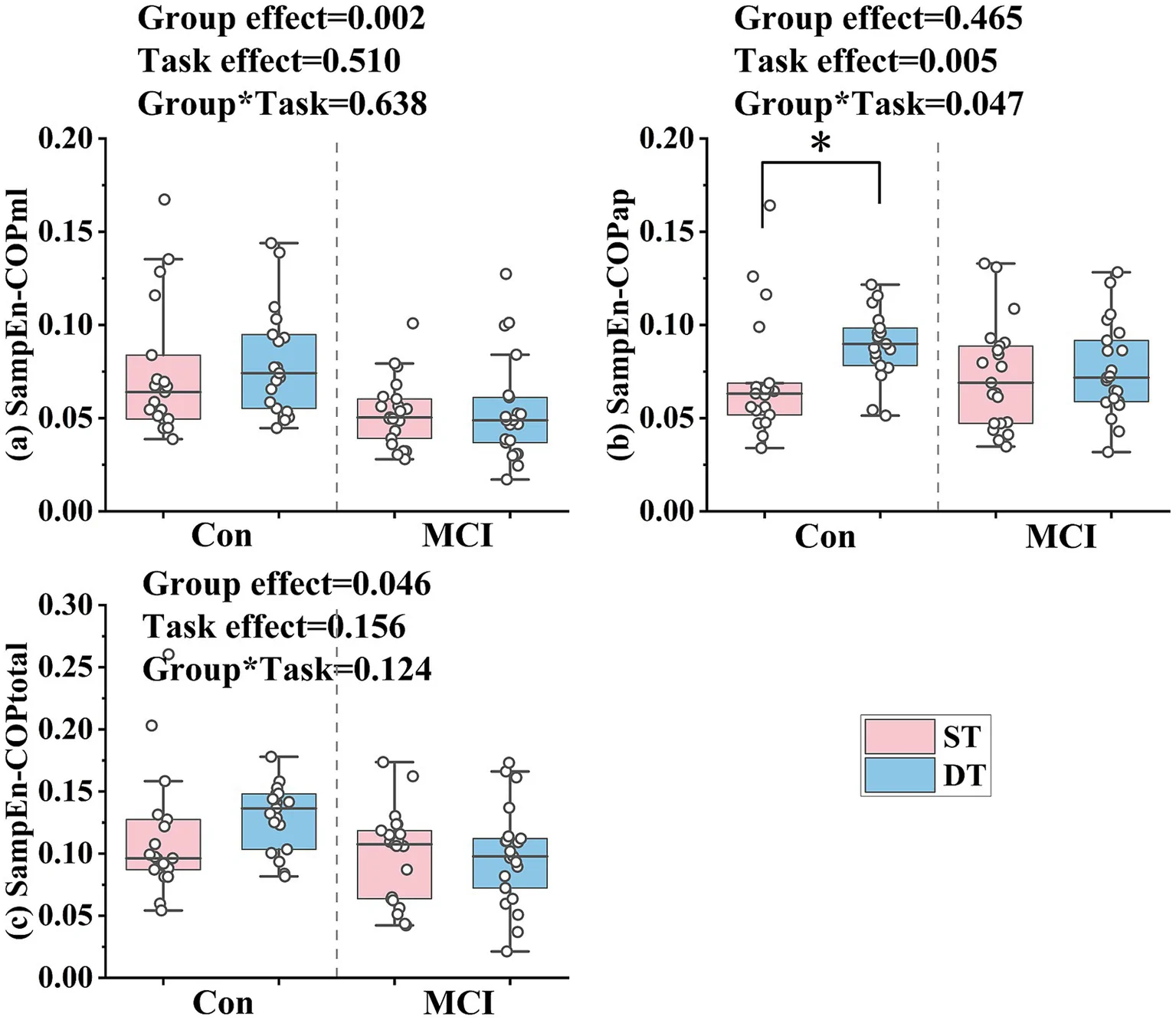
Comparison of COP sample entropy results between control and MCI groups. **(a–c)** Show SampEn-COPml, SampEn-COPap, and SampEn-COPtotal, respectively. SampEn-COPap = sample entropy of center of pressure anterior–posterior displacement; SampEn-COPml = sample entropy of center of pressure medial-lateral displacement; SampEn-COPtotal = the sample entropy of resultant COP displacement. *p < 0.05.

### 3.3 Prefrontal activation levels

Two-way mixed-design ANOVA revealed Group × Task interactions that were significant at the uncorrected level in the DLPFC (95% CI: 0.34 to 14.61, *p* = 0.041, η^2^_p_ = 0.106, q = 0.073) and OFC (95% CI: 0.53 to 19.47, *p* = 0.039, η^2^_p_ = 0.107, q = 0.073). Bonferroni-adjusted *post hoc* analyses showed that, in the MCI group, ΔHbO_2_ was significantly higher under the dual-task condition than under the single-task condition in both the DLPFC (95% CI: 3.33 to 13.17, *p* = 0.002, d = 0.68) and OFC (95% CI: 3.65 to 16.70, *p* = 0.003, d = 0.76). Under the single-task condition, DLPFC ΔHbO_2_ was significantly higher in the control group than in the MCI group (95% CI: 1.42 to 11.34, *p* = 0.013, d = 0.82). The Group × Task interactions were not significant in the FPC (*p* = 0.140, η^2^_p_ = 0.056) or VLPFC (*p* = 0.055, η^2^_p_ = 0.094).

Task main effects were significant at the uncorrected level in the FPC (95% CI: 0.07 to 7.32, *p* = 0.046, η^2^_p_ = 0.101), DLPFC (95% CI: 0.95 to 8.09, *p* = 0.014, η^2^_p_ = 0.147), and OFC (95% CI: 0.44 to 9.91, *p* = 0.033, η^2^_p_ = 0.114), with q = 0.051 for all three effects. ΔHbO_2_ was higher under the dual-task condition than under the single-task condition. No significant task effect was observed in the VLPFC (*p* = 0.051, η^2^_p_ = 0.096). No significant main effects of group were observed in any prefrontal ROI (all *p* ≥ 0.181). Complete ANOVA results and BH-FDR q values are provided in Supplementary Table S2 (Figures 4, 5).

**Figure 4:**
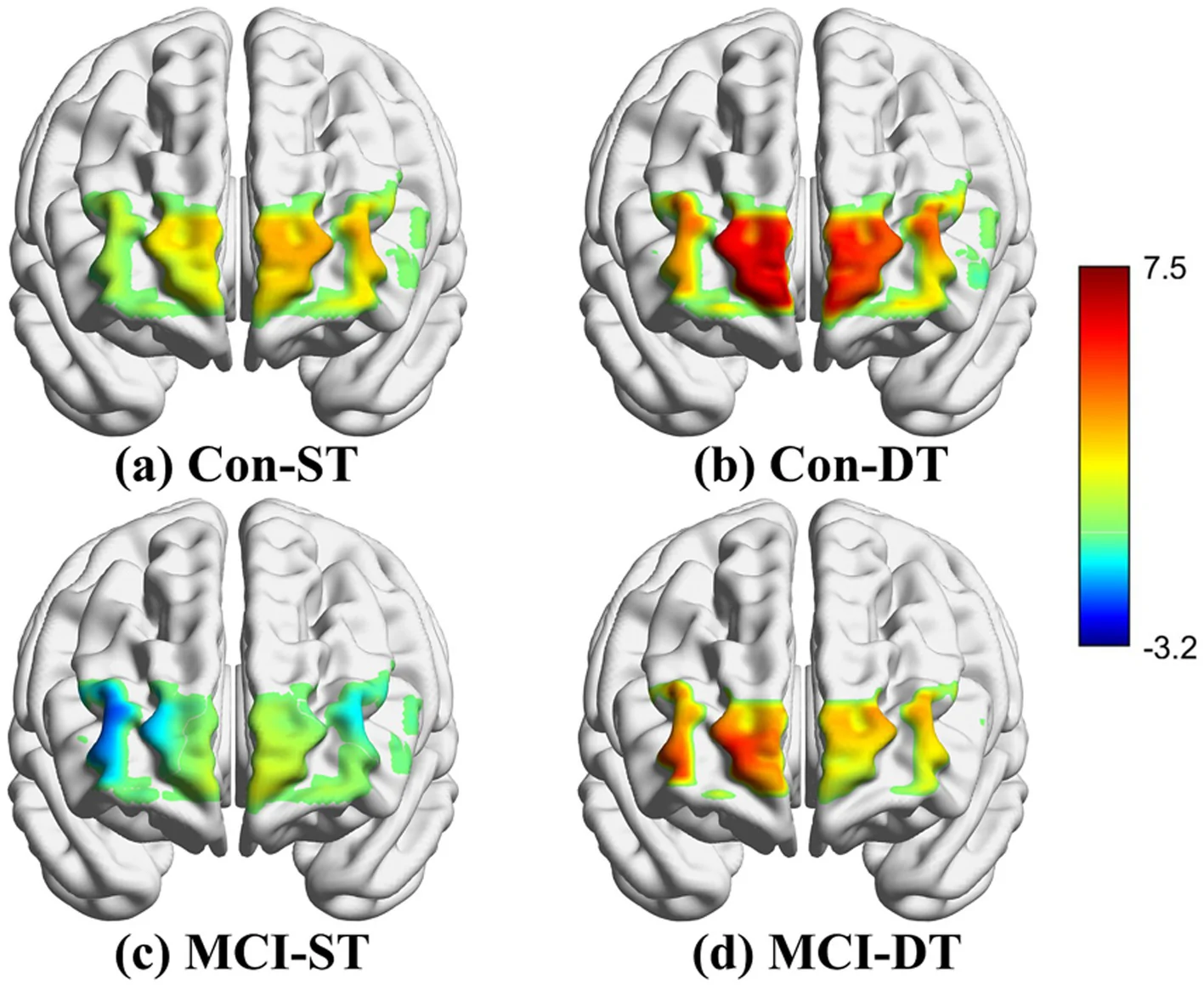
3D plot of PFC activation levels in the control and MCI groups in the single-task and dual-task conditions. **(a)** Represents PFC activation levels in the control group under the single-task condition; **(b)** represents PFC activation levels in the control group under the dual-task condition; **(c)** represents PFC activation levels in the MCI group under the single-task condition; and **(d)** represents PFC activation levels in the MCI group under the dual-task condition. Color mapping of changes in oxyhemoglobin concentration (ΔHbO_2_, μmol/L), color scale range: Blue, inhibition; red, activated. *p < 0.05.

**Figure 5:**
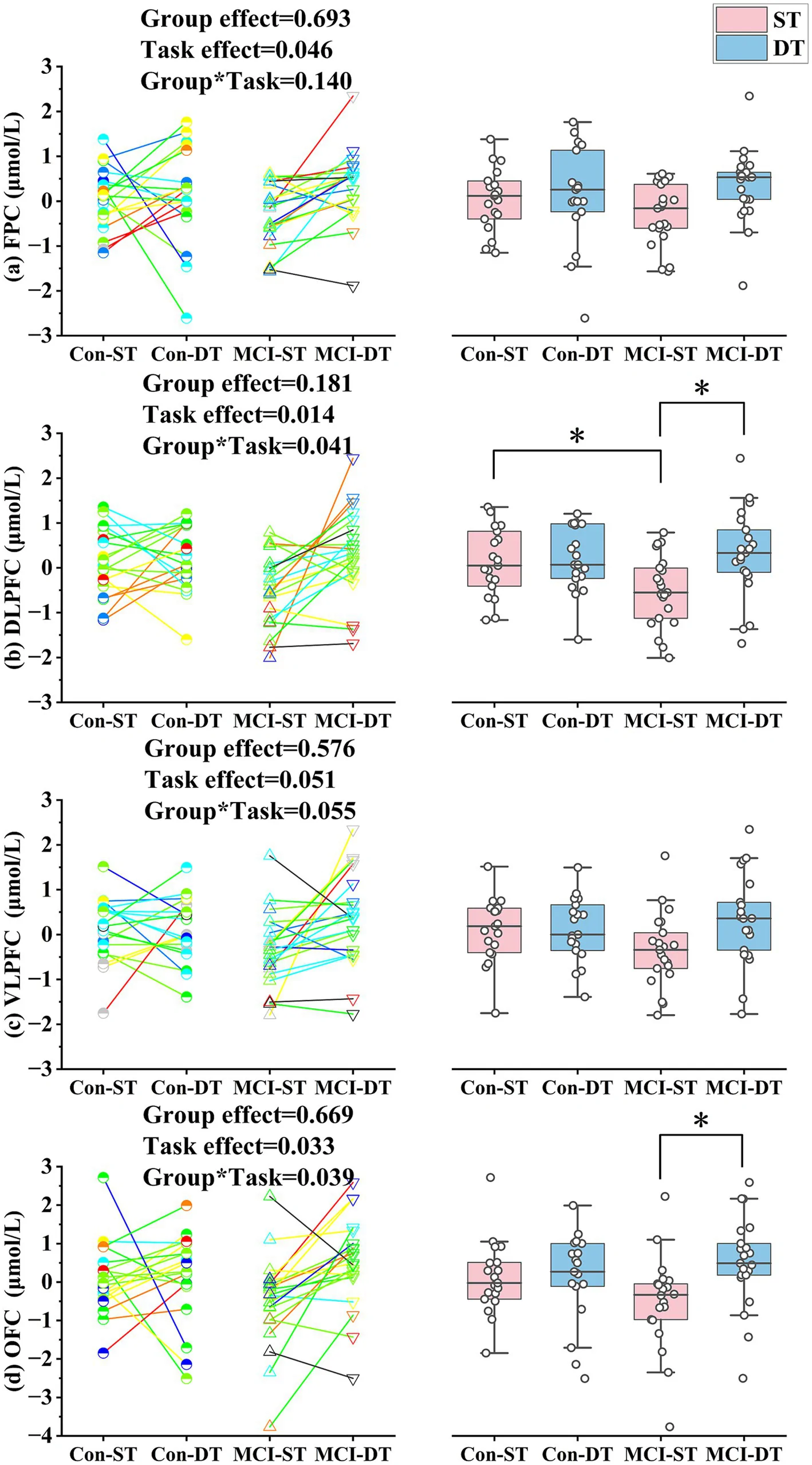
Comparison of PFC activation levels between MCI and control groups under single-task and dual-task conditions. **(a–d)** Represent ΔHbO_2_ in the FPC, DLPFC, VLPFC, and OFC. *, *p* < 0.05.

### 3.4 Exploratory correlations

Exploratory Pearson correlation analyses were performed to examine the relationships between DLPFC and OFC activation (mean ΔHbO_2_) and SampEn-COPap. In the MCI group under the single-task condition, OFC activation showed a negative correlation with SampEn-COPap at the uncorrected level (*r* = −0.469, 95% CI: −0.749 to −0.047, *p* = 0.032, q = 0.256). No significant correlations were observed between DLPFC activation and SampEn-COPap in either group under the single-task or dual-task conditions. Similarly, no significant correlations were found between OFC activation and SampEn-COPap in the control group under either task condition or in the MCI group under the dual-task condition (all *p* ≥ 0.515). Complete correlation coefficients and BH-FDR q values are provided in Supplementary Table S3.

## 4 Discussion

Our findings partially supported the hypothesis. Older adults with MCI showed lower medial–lateral control complexity and poorer cognitive task performance than cognitively intact older adults. However, the expected lower PFC activation was not observed. Instead, DLPFC and OFC activation increased from the single-task condition to the dual-task condition in the MCI group, although the Group × Task interactions did not remain significant after FDR correction. Exploratory correlation analyses did not reveal consistent associations between PFC activation and postural control complexity.

Older adults with MCI showed lower cognitive task accuracy than cognitively intact older adults, and accuracy further decreased under the dual-task condition compared with the single-task condition. However, accuracy DTC and response rate DTC did not differ between groups, indicating that poorer overall cognitive performance in the MCI group did not correspond to greater dual-task costs in either accuracy or response rate. During dual-task walking, serial subtraction performance has been reported to improve relative to single-task performance despite concurrent deterioration in gait, possibly because more attention was allocated to the cognitive task (Fallahtafti et al., 2021). Thus, the lower accuracy observed in the MCI group may reflect poorer overall cognitive performance rather than greater susceptibility to dual-task interference in accuracy. Dual-task performance depends on the allocation of limited attentional and executive resources, which may be less efficiently coordinated in older adults with MCI when concurrent task demands increase (Wang et al., 2022). These findings support the need to consider cognitive performance alongside postural outcomes when evaluating dual-task performance.

SampEn-COPap was higher under the dual-task condition than under the single-task condition across groups. The study results also revealed a Group × Task interaction at the uncorrected level in SampEn-COPap, with significantly higher values under the dual-task condition than under the single-task condition in the control group. The control group also showed an increased response rate under the dual-task condition. However, accuracy was lower under the dual-task condition overall, suggesting that the higher response rate reflected more frequent responding rather than better cognitive performance. Studies have reported lower COP regularity when attention is diverted from postural control by a concurrent cognitive task and have interpreted this pattern as greater reliance on automatic postural control (St-Amant et al., 2020; Donker et al., 2007). The increase in SampEn-COPap in the control group may reflect greater reliance on automatic postural control under dual-task demands. At the same time, the higher response rate reflects more frequent cognitive responses, but not necessarily greater priority to the cognitive task. In contrast, SampEn-COPap did not increase significantly in the MCI group, suggesting a more limited shift toward automatic postural control during dual-task standing.

Moreover, group differences were observed for SampEn-COPml and SampEn-COPtotal, but only the group effect for SampEn-COPml remained significant after FDR correction. Older adults with MCI showed lower complexity of the medial–lateral and resultant COP displacement time series. One possible explanation is that reduced complexity reflects a loss of flexibility within the postural control system. Reduced postural complexity in older adults has been associated with impaired sensory function and a diminished ability to adapt postural control when additional cognitive demands are imposed (Manor et al., 2010). Lower postural complexity has been interpreted as reflecting more constrained postural coordination and reduced redundancy, which may limit the adaptability of postural control (Haid and Federolf, 2018). Lower medial–lateral SampEn has also been reported in older adults with MCI compared with cognitively intact controls under a challenging standing condition (Thammachat et al., 2026). These findings suggest that reduced postural complexity in MCI may reflect less flexible postural regulation, potentially limiting adaptation to changing sensory or cognitive demands. Postural complexity may provide complementary information about the flexibility and temporal organization of postural control beyond conventional sway measures (Kędziorek and Błażkiewicz, 2020). These SampEn-COP differences characterize postural control in MCI, but their relevance to fall risk or rehabilitation has not yet been established.

In the FPC, DLPFC, and OFC, task main effects were observed at the uncorrected level, with greater activation under the dual-task condition than under the single-task condition. This finding is consistent with previous studies reporting increased prefrontal cortex activation during dual-task walking, which may reflect the recruitment of additional cognitive resources to maintain postural stability (Ross et al., 2021). In this study, the greater activation under the dual-task condition may similarly reflect increased prefrontal involvement in response to the additional cognitive demand.

Our results showed Group × Task interactions at the uncorrected level in the DLPFC and OFC, with ΔHbO_2_ significantly higher under the dual-task condition than under the single-task condition in the MCI group. The increased activation in the MCI group may indicate greater recruitment of neural resources to support concurrent cognitive and postural demands. Capacity-Sharing Theory proposes that concurrent tasks compete for limited cognitive resources (Lacour et al., 2008). This competition may increase the need for prefrontal involvement when cognitive and postural demands must be managed simultaneously. Another possible explanation for our results comes from previous findings showing that increased cognitive and postural demands can elicit compensatory increases in PFC activation in older adults (Chen et al., 2022). However, increased PFC activation does not necessarily indicate successful compensation, and greater neural recruitment may also reflect increased neural effort when task demands become more challenging. In this study, greater cortical recruitment in the MCI group was not accompanied by a corresponding increase in anterior–posterior COP complexity. Accordingly, the increased DLPFC and OFC activation may reflect additional prefrontal recruitment in response to dual-task demands, but the present behavioral findings do not provide clear evidence that this recruitment represented successful compensation.

Exploratory correlation analysis showed a negative association between OFC activation and SampEn-COPap in the MCI group under the single-task condition at the uncorrected level. Greater COP regularity has been associated with greater attentional involvement in postural control, whereas increased SampEn under cognitive dual-task conditions has been interpreted as greater reliance on automatic postural control (Donker et al., 2007; Richer and Lajoie, 2020). Accordingly, the present relationship may indicate that greater OFC involvement in older adults with MCI is associated with a more regular pattern of postural control, potentially reflecting greater attentional involvement. Given its exploratory nature, this isolated finding should be interpreted cautiously. The modest sample size may also have limited the detection of smaller effects and the generalizability of the findings, and the present findings should be confirmed in larger samples.

## 5 Study limitations

First, fNIRS data were acquired using a 22-channel system with optodes positioned over the PFC, and no short-separation channels were included to account for superficial hemodynamic contributions. Accordingly, the findings may primarily reflect prefrontal involvement in dual-task regulation rather than broader cortical involvement. However, the focus on the PFC is reasonable given its central role in executive control and attention allocation processes involved in dual-task postural performance. Second, although MCI classification incorporated clinical and functional criteria in addition to MoCA screening, the lack of a comprehensive neuropsychological assessment may have limited the detailed characterization of cognitive impairment. Finally, although serial subtraction has been shown to affect standing balance (Manor et al., 2010), it represents a specific quantitative cognitive task, and its sustained attention and working-memory demands may not fully reflect those required during non-quantitative dual tasks encountered in daily life. As this was a cross-sectional study, longitudinal and intervention studies are needed to further examine postural control complexity and PFC activation during dual-task performance.

## 6 Conclusion

Older adults with MCI exhibited lower medial–lateral postural control complexity than cognitively intact older adults while dual-task standing increased anterior–posterior postural control complexity. Task-related differences in DLPFC and OFC activation were also observed between groups at the uncorrected level, providing preliminary evidence of altered prefrontal responses during dual-task performance in older adults with MCI. Future research should further explore the contribution of distributed brain networks to postural control.

## Data Availability

The original contributions presented in the study are included in the article/Supplementary material, further inquiries can be directed to the corresponding author.
